# Dual Paraneoplastic Endocrine Syndromes Heralding Onset of Extrapulmonary Small Cell Carcinoma: A Case Report and Narrative Review

**DOI:** 10.3389/fendo.2018.00170

**Published:** 2018-04-18

**Authors:** Jill B. Feffer, Natalia M. Branis, Jeanine B. Albu

**Affiliations:** Division of Endocrinology, Diabetes and Metabolism, Icahn School of Medicine at Mount Sinai, Mount Sinai St. Luke’s and West Hospital Center, New York, NY, United States

**Keywords:** ectopic Cushing’s syndrome, humoral hypercalcemia, PTH-related peptide, neuroendocrine carcinoma, androgen deprivation therapy, extrapulmonary small cell carcinoma

## Abstract

**Objective:**

Extrapulmonary small cell carcinoma (EPSCC) is rare and frequent metastases at presentation can complicate efforts to identify a site of origin. In particular, SCC comprises <1% of prostate cancers and has been implicated in castration resistance.

**Methods:**

Clinical, laboratory, imaging, and pathology data are presented.

**Results:**

A 56-year-old man with locally advanced prostate adenocarcinoma on androgen deprivation therapy presented with a clogged nephrostomy tube. Laboratory results included calcium 13.8 mg/dL (8.5–10.5 mg/dL), albumin 3.6 g/dL (3.5–5 mg/dL), and potassium 2.8 mmol/L (3.5–5.2 mmol/L). Hypercalcemia investigation revealed intact PTH 19 pg/mL (16–87 pg/mL), 25-OH vitamin D 15.7 ng/mL (>30 ng/mL), and PTH-related peptide (PTHrP) 63.4 pmol/L (<2.3 pmol/L). Workup for hypokalemia yielded aldosterone 5.3 ng/dL (<31 ng/dL), renin 0.6 ng/mL/h (0.5–4 ng/mL/h), and 6:00 a.m. cortisol 82 µg/dL (6.7–22.6 µg/dL) with ACTH 147 pg/mL (no ref. range). High-dose Dexamethasone suppression testing suggested ACTH-dependent ectopic hypercortisolism. Contrast-enhanced CT findings included masses in the liver and right renal pelvis, a heterogeneous enlarged mass in the region of the prostate invading the bladder, bilateral adrenal thickening, and lytic lesions in the pelvis and spine. Liver biopsy identified epithelioid malignancy with Ki proliferation index 98% and immunohistochemical staining positive for synaptophysin and neuron-specific enolase, compatible with high-grade small cell carcinoma. Staining for ACTH was negative; no stain for CRH was available. Two weeks after chemotherapy, 6:00 a.m. cortisol normalized and CT scans showed universal improvement.

**Conclusion:**

Extensive literature details paraneoplastic syndromes associated with SCC, but we report the first case of EPSCC diagnosed due to onset of dual paraneoplastic syndromes.

## Introduction

Extrapulmonary small cell carcinoma (EPSCC) is an uncommon presentation of SCC. Its aggressive behavior frequently results in early metastases, which can complicate attempts to identify the site of origin. Extensive literature details paraneoplastic syndromes associated with neuroendocrine neoplasms, such as SCC (1–52), but there are no reports of co-occurring ectopic hypercortisolism and humoral hypercalcemia at initial diagnosis.

Additionally, SCC is an unusual, aggressive variant of prostate carcinoma and has been implicated in castration resistance (53). SCC may be diagnosed at the time of initial diagnosis of prostate cancer or may arise as a secondary malignancy following androgen deprivation therapy (ADT) to treat the more common prostate adenocarcinoma (49, 54).

## Methods

Written informed consent was obtained from the patient for the publication of this case report. Clinical, laboratory, imaging, and pathology data are presented. We performed a search of the PubMed database through October 2017 for studies addressing paraneoplastic syndromes associated with neuroendocrine tumors, with particular emphasis on prostate carcinoma, Cushing’s syndrome, and hypercalcemia. Relevant cited articles were also retrieved and non-English articles were translated when possible.

## Case Report

A 56-year-old man with prostate adenocarcinoma presented to the emergency department with clogged nephrostomy tubes. He had initially been diagnosed with Gleason 3 + 4 prostate adenocarcinoma 5 years prior to admission, then was lost to follow-up for 4 years. Repeat biopsy at that time reportedly revealed Gleason 5 + 5 prostate adenocarcinoma. The full pathology is not available but to the best of our knowledge, no neuroendocrine component was reported after either biopsy. At this time, the patient had local progression of disease to the rectum and bladder and bilateral hydroureteronephrosis so he underwent channel transurethral resection of his prostate and was started on daily oral bicalutamide and monthly leuprolide injections, which he was still taking at the time of admission. The patient was also referred for external beam radiation, but did not establish care with radiation oncology.

Review of systems was positive for constipation, weight loss, anorexia, and imbalance. Physical exam was notable for cachexia. Admission laboratory evaluation showed calcium 13.8 mg/dL (8.5–10.5 mg/dL), albumin 3.6 mg/dL (3.5–5 mg/dL), and potassium 2.8 mmol/L (3.5–5.2 mmol/L). Two months prior to admission, calcium had been reported 9.1 mg/dL (8.5–10.5 mg/dL) with no albumin reported and potassium 3.9 mmol/L (3.5–5.3 mmol/L). Further biochemical testing to determine the etiology of this patient’s hypercalcemia found intact PTH 19 pg/mL (16–87 pg/mL), 25-OH vitamin D 15.7 ng/mL (>30 ng/mL), and PTH-related peptide (PTHrP) 63.4 pmol/L (<2.3 pmol/L). Pamidronate was administered. Six days later, the patient experienced transient hypocalcemia to 6.6 mg/dL (8.5–10.5 mg/dL) with albumin 3.3 mg/dL (3.5–5 mg/dL). Hypocalcemia resolved the next day and the patient has remained normocalcemic for 8 months post-treatment.

Biochemical investigation in search of the underlying cause of refractory hypokalemia as low as 2.4 mmol/L (3.2–5.2 mmol/dL) revealed aldosterone 5.3 ng/dL (<31 ng/dL), renin 0.6 ng/mL/h (0.5–4 ng/mL/h), and 6:00 a.m. cortisol 82 µg/dL (6.7–22.6 µg/dL) with ACTH 147 pg/mL (no ref. range). After high-dose Dexamethasone suppression testing with 8 mg administered at midnight, 6:00 a.m. cortisol remained 62 µg/dL (6.7–22.6 µg/dL) with ACTH 115 pg/mL (no ref. range), compatible with ectopic ACTH-dependent hypercortisolism. Ketoconazole was initiated along with Spironolactone to address refractory hypokalemia as well as resistant hypertension (HTN) already treated with Enalapril, Carvedilol, and Amlodipine. The patient’s hemoglobin A1c on admission was 5.8% (40 mmol/mol) (4.2–5.9%, 22–41 mmol/mol), signifying prediabetes. New-onset severe hyperglycemia as high as >400 mg/dL during the second week of this hospitalization was managed with Glargine and Lispro insulins.

**Figure 1:**
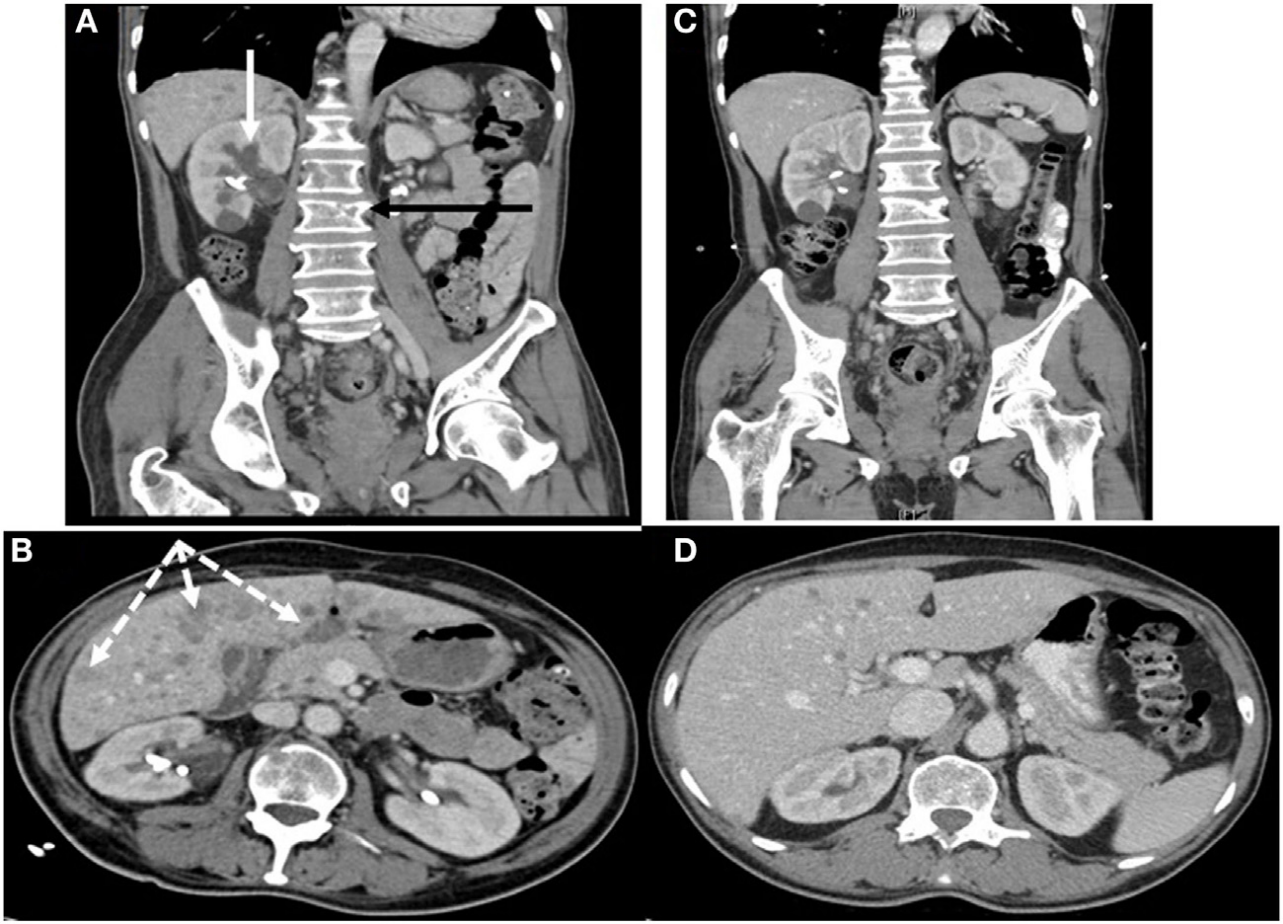
Contrast-enhanced CT scans of chest, abdomen, and pelvis **(A,B)** showing renal, hepatic, and osseous metastases before chemotherapy **(C,D)** 2 weeks afterward showing significant interval improvement. **(A)** Hypoattenuated masses in the right renal pelvis and ureter (solid white arrow) and lytic lesions in the spine (black arrow). **(B)** Multiple hypoattenuated hepatic masses (dashed white arrows).

**Figure 2:**
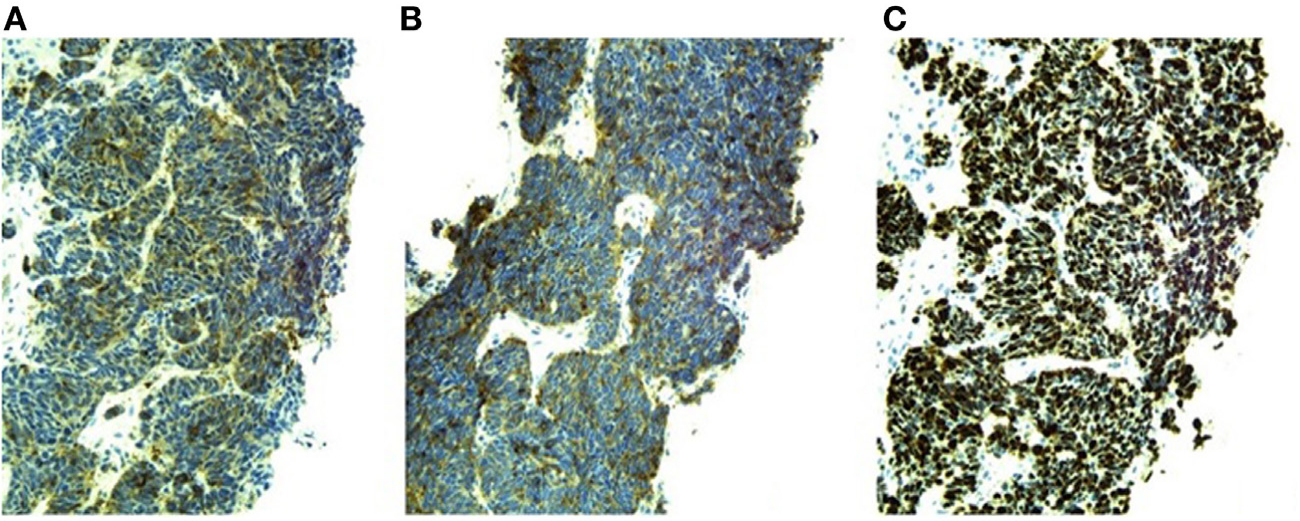
Positive immunohistochemical stains and Ki-67 proliferation index of liver biopsy sample (20× magnification). **(A)** Neuron-specific enolase. **(B)** Synaptophysin. **(C)** Ki-67 proliferation index 98%.

Pituitary MRI would have been a reasonable next step but was not performed. Given this patient’s history of malignancy on hormonal therapy, subacute clinical presentation and new diagnosis of PTHrP-mediated hypercalcemia, our suspicion for an ectopic source of ACTH-dependent hypercortisolism was very high so localizing imaging was performed urgently. Contrast-enhanced CT scans of the chest, abdomen, and pelvis detected multiple hypoattenuated masses in the liver up to 3.3 cm in diameter, subcentimeter pleural nodules, multiple masses in the right renal pelvis and ureter up to 1.2 cm in diameter, necrotic lymph nodes, a heterogeneous enlarged mass in the region of the prostate invading the bladder, bilateral adrenal thickening, and lytic lesions in the pelvis and lumbar spine (see Figures 1A,B). Medical oncology was consulted when the biopsy of a liver lesion identified epithelioid malignancy with cords and nests of small to medium size cells with high N/C ratio, salt and pepper chromatin, evident mitoses and numerous apoptotic bodies, Ki proliferation index 98%, and immunohistochemistry (IHC) staining positive for synaptophysin, neuron-specific enolase, CD56, cytokeratins CAM 5.2 and AE1/AE3, and focally for CK20, consistent with high-grade small cell neuroendocrine carcinoma (see Figure 2). IHC staining was negative for PSA, PTH, and ACTH. Stains for PTHrP and CRH were not available.

Five days after initiation of treatment with Carboplatin and Etoposide chemotherapy targeting EPSCC, the patient’s 6:00 a.m. cortisol decreased to 10 µg/dL (6.7–22.6 µg/dL) so Ketaconazole and Spironolactone were discontinued. CT scans obtained 2 weeks after chemotherapy initiation documented diminution or resolution of all prior findings (see Figures 1C,D). Repeated CT scans 7 months later showed an increase in number of hepatic lesions, a stable mass in the region of the prostate invading the bladder, stable pelvic lymphadenopathy, and sclerotic changes in known spinal and pelvic metastases without new bone lesions. The next month, now 8 months after chemotherapy initiation and following five more cycles of Carboplatin and Etoposide plus two cycles of Irinotecan, his potassium level was 3.5 mmol/L (3.2–5.2 mmol/dL) and calcium 9.6 mg/dL (8.5–10.5 mg/dL) with albumin 4.2 mg/dL (3.5–5 mg/dL). He was taking Lisinopril, Carvedilol, and Hydrochlorothiazide at an oncology appointment 8 months after chemotherapy initiation.

## Discussion

### Epidemiology of EPSCC

The age-adjusted incidence rate (AAIR) of EPSCC is estimated to be 3.5/1,000,000 person-years in the United States, only 1/22 that of pulmonary SCC (55). Such low incidence limits available literature. Papers from North America, Asia, and Europe report conflicting prevalence data, but agree on the overall most common categories of primary sites: gastrointestinal, genitourinary, gynecologic, and head and neck (see Table 1).

**Table 1:** Extrapulmonary small cell carcinoma: % frequency of sites of origin.

Reference	Breast	GI	GU (prostate)	Gyn	H&N	Unknown	Other
(55)	0	55.6	11.1 (0)	11.1	0	22.2	0
(56)	6.25	31.25	18.75 (12.5)	18.75	6.25	18.75	0
(57)	0	29.2	29.2 (0)	29.2	8.33	0	4.67 (thymus)
(58)	9	20	18 (not reported)	11	10	30	2 (thymus)
(59)	0	67.3	0	9.6	19.2	0	3.9 (thyroid, pleura)
(60)	0	56	10 (5)	20	8	0	6 (bone, retroperitoneal mass)
(61)	0	30	11 (not reported)	30	19	10	0
(62)	3.6	14.3	28.6 (14.3)	17.8	21.4	14.3	0
(63)	10.3	33	10.9 (5.6)	7.9	9.1	4	0.7 (skin), 2.5 (thyroid), 7 (heart, mediastinum, pleura, unspecified in chest), 14.6 (unspecified)
(64)	0	39	18 (3)	14	6	14	9 (skin)
(65)	0	81.0	9.5 (4.75)	0	0	4.75	4.75 (thymus)
(66)	2	15	26 (not reported)	6	11	40	0
(67)	1.9	22.6	32.1 (10.7)	30.8	8.8	3.8	0

*GI, gastrointestinal; GU, genitourinary; Gyn, gynecologic; H&N, head and neck*.

Genitourinary EPSCC includes malignancies that originate in the prostate as well as in the kidney, urinary bladder, urinary tract, and other reproductive organs. In particular, SCC has been noted to occur in patients previously diagnosed with prostate adenocarcinoma who were being treated with ADT such as our patient. For example, a retrospective chart review of 83 patients diagnosed with prostatic SCC over a 20-year period found 67% of the cohort had been previously diagnosed with prostate adenocarcinoma, while histologic diagnoses of SCC were 64% mixed SCC and adenocarcinoma rather than pure SCC (68).

Existing data regarding the clinical and laboratory characteristics of patients diagnosed with neuroendocrine prostate carcinoma is limited by low prevalence but does demonstrate some common features. Two retrospective studies of the SEER database describe the population diagnosed with prostatic SCC as being predominately older than 80 years of age and Caucasian. The majority of patients also exhibited distant metastases and low PSA level at the time of initial diagnosis (68, 69). AAIR of this entity has been estimated to be 0.582/1,000,000 person-years in 2011 (69), while SCC represented only 0.06% of all prostate cancers diagnosed between 1973 and 2008 in the United States (68).

### Hypothesized Mechanisms for Post-ADT Prostatic Neuroendocrine Cancer

Postulated mechanisms of the concurrent or subsequent diagnosis of prostate adenocarcinoma and SCC include the reversal of quiescence in neuroendocrine cell nests routinely present in adenocarcinoma by ADT or independent development from shared multipotent progenitor cells (70, 71). Therapeutic targets being explored include: (1) the tyrosine kinase AURKA found to be overexpressed by prostate adenocarcinoma which includes a neuroendocrine component, (2) the oncogene MYCN, which is also overexpressed, (3) the silencing transcription factor gene REST, found to be underexpressed, (4) the gene Rb encoding a regulator of the cell cycle which is lost, (5) the metalloproteinase inhibitor TIMP-1 that is overexpressed, (6) the cell adhesion protein CD44 that is expressed only in the neuroendocrine component of mixed prostate malignancies, and (7) the aberrant fusion product of genes encoding the serine protease TMPRSS2 and the zinc finger transcription factor ERG, which are both overexpressed in neuroendocrine prostate malignancies (71–73).

### Treatment of EPSCC

The North American Neuroendocrine Tumor Society issued management guidelines for EPSCC in 2010 (74). These guidelines are recommended for all cases, regardless of site of origin. The current standard of care is to treat ESPCC similarly to pulmonary SCC: attempt debulking with or without adjunctive radiation treatment for local or locoregional disease, otherwise systemic treatment with platinum-based chemotherapy and Etoposide is first-line therapy for metastatic disease at presentation. Findings vary regarding whether significant differences in response to treatment modalities, progression-free survival, and overall survival exist between pulmonary SCC and EPSCC and between EPSCC of different sites of origin, but most studies agree on improved survival in EPSCC and in younger patients with limited rather than metastatic disease (55, 65, 66, 75).

### Ectopic Endocrine Syndromes Associated With Neuroendocrine Neoplasms

Neuroendocrine neoplasms, most commonly pulmonary SCC and carcinoid tumors, have been known to produce many ectopic hormones, including ACTH and CRH. These typically trigger rapid onset of objective signs of hypercortisolism without Cushingoid features on physical exam. Such features include HTN, insulin resistance and hyperglycemia, hyperpigmentation due to increased expression of the alpha-subunit of ACTH or increased precursor peptide POM-C expression, and hypokalemia due to activity at the mineralocorticoid receptor, which also results in suppressed serum renin levels. These patients typically present with cachexia, weight loss, and easy bruising due to severe catabolism and are known to be at increased risk for steroid-induced psychosis. This atypical cohort constitutes up to 20% of all cases of Cushing’s syndrome (1).

Extrapulmonary neuroendocrine neoplasms, including EPSCC, can produce a variety of ectopic hormones to cause other kinds of paraneoplastic syndromes as noted in case reports. For example, prostatic undifferentiated carcinoma has been linked to both SIADH (2, 3) and atypical myasthenia gravis due to Lambert–Eaton syndrome (4). Additional case reports support the postulated ectopic secretion of ADH with positive IHC staining in prostatic SCC (5). Paraneoplastic SIADH has also been associated with SCC of the nasal cavity and ethmoid sinuses (6–8). Neuroendocrine neoplasms of the GI tract, including the esophagus, liver, gall bladder, pancreas, and colon have likewise been linked to SIADH, humoral hypercalcemia of malignancy, and anti-Hu sensory neuropathy (9–16). Similarly, SCC of the vagina, cervix, endometrium, ovary, and breast have been documented to show IHC positivity for ACTH, the ACTH precursor peptide POM-C, PTH, and PTHrP (17–25). Case reports and series of patients with prostatic SCC have documented the occurrence of ectopic hypercortisolism among only 26 patients in 24 papers, since 1973 (see Table 2). Similarly, humoral hypercalcemia due to ectopic PTHrP has been reported in prostate adenocarcinoma (26, 27), while an *in vitro* study localized PTHrP expression to nests of neuroendocrine cells within prostate adenocarcinoma (28). Again, the relative rarity of these conditions limits data available beyond case series.

**Table 2:** Reports of ectopic hypercortisolism due to prostatic neuroendocrine cancer.

Reference	Histology	Immunohistochemistry staining positive?	Prior diagnosis of prostate cancer?	Prior hormonal therapy?	Presenting symptoms	Hypokalemia present at diagnosis	Hypertension (HTN) present at diagnosis	Hyperglycemia present at diagnosis
(29)	UD	Not reported	Yes	Yes	Peripheral edema, altered mental status	Yes	Yes	Yes
(30)	UD	Not reported	Yes	Yes	Peripheral edema, weakness, polyuria, bone pain, weight gain; Cushingoid appearance	No	Yes	Yes
(31)	SCC	Yes, ACTH	Yes	No	Weakness, peripheral edema, urinary obstruction	Yes	Yes	Yes
(32)	UD	Yes, ACTH	Yes	Yes	Hematuria, acute psychosis; Cushingoid appearance	Yes	Yes	Yes
(33)	Mixed	Yes, ACTH	Yes	Yes	Not reported	Not reported	Not reported	Not reported
(34)	SCC	Yes, CRH	No	No	Weight loss	Yes	No	Yes
(35)	Carcinoid	Yes, ACTH	No	No	Hematuria, nocturia, oliguria	Yes	No	Yes
(36)	Mixed	Yes, ACTH	No	No	Hematuria, bone pain	No	No	No
(37)	SCC	Yes, CRH	No	No	Abdominal pain, weakness, weight loss, urinary obstruction	Yes	Yes	No
(38)	UD	Yes, ACTH	No	No	Back pain; Cushingoid appearance	Yes	Yes	Yes
(39)	Mixed	Not reported	Yes	Yes	Bone pain, urinary obstruction, peripheral edema	Yes	No	Yes
(40)	UD	Not reported	Yes	Yes	Altered mental status	Yes	Yes	Yes
(41)	Mixed	Yes, ACTH	Yes	Yes	Nocturia, hematuria, bone pain, peripheral edema	Yes	Yes	Yes
(42)	1 mixed, 1 SCC	Not reported	Yes	Yes	Both: peripheral edema; 1 weakness, 1 fatigue	Yes	Not reported	Not reported
(43)	2 SCC	Not reported	Yes	Yes	Both: hematuria; 1 bone pain, hematuria, urinary obstruction, 1 polyuria, weakness, fatigue, altered mental status	Yes	Yes	Yes
(44)	SCC	Yes, ACTH	No	No	Weakness, weight loss, peripheral edema	Yes	Yes	Yes
(45)	SCC	Yes, ACTH	No	No	Urinary obstruction, weakness, peripheral edema	Yes	Yes	Yes
(46)	SCC	Yes, ACTH	No	No	Peripheral edema, weakness	Yes	Yes	Yes
(47)	SCC	Not reported	Yes	Yes	Abdominal pain, bone pain, weight loss	No	Yes	Yes
(48)	SCC	Yes, ACTH	No	No	Confusion, weight gain, peripheral edema, increased urinary tract infections	Yes	Yes	Yes
(49)	SCC	Not reported	Yes	Yes	Bone pain, fatigue, new HTN, hyperglycemia, hypokalemia, and skin hyperpigmentation	Yes	Yes	Yes
(50)	SCC	Not reported	No	No	Back pain, edema	Yes	Yes	Yes
(51)	SCC	Yes, ACTH	No	No	Weight loss, productive cough, muscle weakness, urinary incontinence, constipation, delirium	Yes	Yes	Yes
(52)	Mixed	Yes, ACTH	No	No	Weakness, labile mood, peripheral edema, weight gain	Yes	Yes	No

*SCC, small cell carcinoma; UD, undifferentiated carcinoma (small cell or large cell); Mixed, SCC + adenocarcinoma*.

Although the primary site of our patient’s EPSCC could not be definitively identified, we believe it is of GU origin given the patient’s history of taking ADT and the epidemiology of EPSCC as discussed above. The decision to biopsy a hepatic lesion was made by the interventional radiologist who performed the procedure, and the consulting medical oncologist did not require further data from other sites to form a treatment plan so no additional biopsy was performed. Based on global improvement on post-treatment CT scans, it is reasonable to conclude that all lesions are the same SCC as diagnosed in the liver. We suspect our patient’s SCC liver biopsy sample tested was negative in IHC staining for ACTH because it was instead producing CRH or POM-C; however, other possible explanations cannot be ruled out. These include sampling error of biopsy leading to a false negative result, mosaicism among individual cells or metastatic sites of this malignancy, or a coexisting distinct malignancy producing ACTH.

## Concluding Remarks

To the best of our knowledge, we present the first reported case of EPSCC diagnosed due to onset of dual paraneoplastic syndromes. Overall, ectopic endocrine syndromes due to neuroendocrine neoplasms are rare, but can be important markers of onset or progression of malignancy. Moreover, hormonal excess causes significant morbidity in its own right.

This particular case also highlights the risk of development of aggressive neuroendocrine carcinoma after hormonal treatment of prostate adenocarcinoma. Further research is indicated to elucidate the pathophysiology of post-ADT EPSCC and determine clinicopathologic characteristics that can predict which patients are most at risk. Such information would hopefully facilitate development of screening guidelines for this disease.

## Author Contributions

JF drafted the original manuscript and created all figures and tables. NB and JA contributed to expansion of the discussion and proofread the manuscript.

## Conflict of Interest Statement

The authors declare that the research was conducted in the absence of any commercial or financial relationships that could be construed as a potential conflict of interest.
